# Emerging Role of Long Non-Coding RNA *SOX2OT* in *SOX2* Regulation in Breast Cancer

**DOI:** 10.1371/journal.pone.0102140

**Published:** 2014-07-09

**Authors:** Marjan E. Askarian-Amiri, Vahid Seyfoddin, Chanel E. Smart, Jingli Wang, Ji Eun Kim, Herah Hansji, Bruce C. Baguley, Graeme J. Finlay, Euphemia Y. Leung

**Affiliations:** 1 Auckland Cancer Society Research Centre, University of Auckland, Auckland, New Zealand; 2 University of Queensland Centre for Clinical Research, Royal Brisbane & Women's Hospital Campus, Herston, Queensland, Australia; University of Alabama at Birmingham, United States of America

## Abstract

The transcription factor *SOX2* is essential for maintaining pluripotency in a variety of stem cells. It has important functions during embryonic development, is involved in cancer stem cell maintenance, and is often deregulated in cancer. The mechanism of *SOX2* regulation has yet to be clarified, but the *SOX2* gene lies in an intron of a long multi-exon non-coding RNA called *SOX2 overlapping transcript* (*SOX2OT*). Here, we show that the expression of *SOX2* and *SOX2OT* is concordant in breast cancer, differentially expressed in estrogen receptor positive and negative breast cancer samples and that both are up-regulated in suspension culture conditions that favor growth of stem cell phenotypes. Importantly, ectopic expression of *SOX2OT* led to an almost 20-fold increase in *SOX2* expression, together with a reduced proliferation and increased breast cancer cell anchorage-independent growth. We propose that *SOX2OT* plays a key role in the induction and/or maintenance of *SOX2* expression in breast cancer.

## Introduction

The *SOX2* gene family (SRY-related HMG-box) encodes a group of transcription factors that are each characterized by the presence of a highly conserved high-mobility group (HMG) domain [1], [2]. *SOX2* genes are also highly conserved [3] and have been extensively studied in embryonic stem cells, especially in early foregut and neural development. They have been found to be expressed in a restricted spatial-temporal pattern and to play a critical role in stem cell biology, organogenesis, and animal development [3]. *SOX2* was also shown to participate in reprogramming of adult somatic cells to a pluripotent stem cell state and has been implicated in tumorigenesis in various organs [4], [5], [6], [7]. Differential expression of *SOX2* is reported in human cancers [8], [9], [10], [11], [12], [13], [14], [15], [16], [17], [18], including breast cancer from cancer patients and breast cancer cell lines [10], [13], [14], [19], [20]. *SOX2* expression has been observed in 43% of basal cell-like breast carcinomas and has been found to be strongly correlated with CK5/6, EGFR, and vimentin immunoreactivity, and to be inversely associated with estrogen and progesterone receptor status, suggesting that *SOX2* plays a role in conferring a less differentiated phenotype in these tumors [21], [22]. Other groups have reported *SOX2* expression in a variety of early stage postmenopausal breast carcinomas and lymph nodes metastases, suggesting that *SOX2* may play an early role in breast carcinogenesis and that high expression may promote metastatic potential [14]. Expression of *SOX2* is up-regulated in a breast cancer cell line grown in a three-dimensional collagen scaffold which partially simulates *in vivo* conditions [23].

Stem cell-like features may be functionally demonstrated *in vitro* by the ability of cancer stem cells to grow as mammospheres in non-adherent/serum-free stem cell conditions [24]. Over-expression of *SOX2* increased mammosphere formation, and the effect was dependent on continuous *SOX2* expression; furthermore, *SOX2* knockdown prevented mammosphere formation and delayed tumor formation in xenograft tumor initiation models [25].

**Figure 1:**
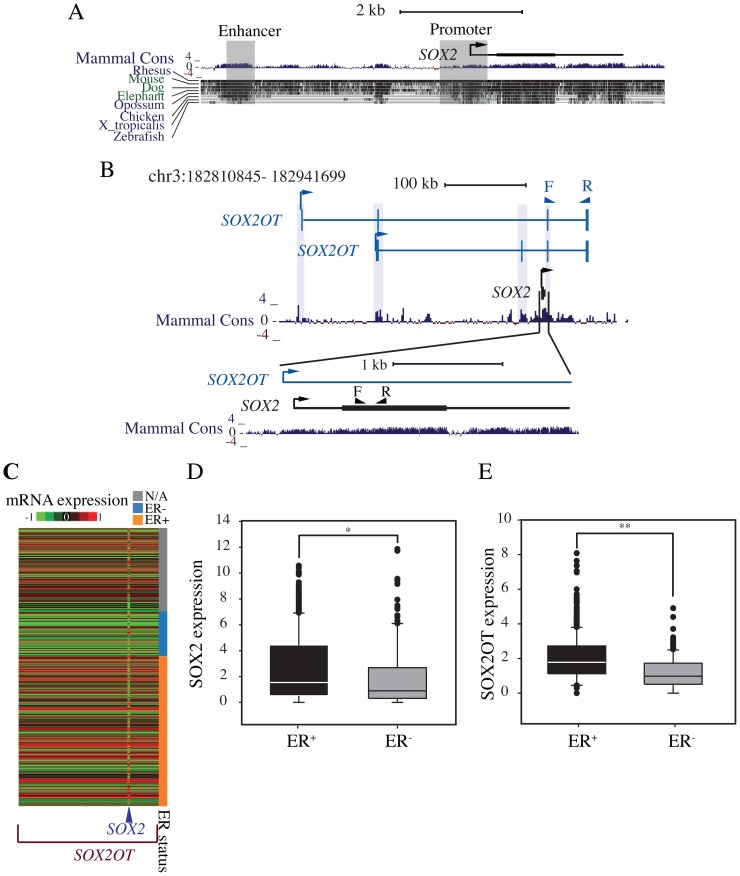
Schematic genomic organization of *SOX2* and *SOX2OT* and their expression patterns in breast cancer. A) The genomic context of *SOX2* and its upstream region. The gray boxes show the proposed promoter and enhancer regions [26], [27]. B) The genomic context of *SOX2* and *SOX2OT*, derived from the UCSC browser. *SOX2* is located in an intron of *SOX2OT*. The triangles above each gene show the location of primers used in qRT-PCR. The *SOX2* region is enlarged, and the direction of gene transcription shown with arrows. The phylogenetic conservation of each region is shown below the gene diagram (Mammal Cons). Vertical rectangular shading shows the conserved exonic regions of *SOX2OT*. The locations of these genes are adopted from UCSC genome browser March 2006 (NCBI36/hg18). C) Heat map showing the expression of *SOX2* and *SOX2OT* in breast cancer samples analyzed by TCGA. It covers the expression of *SOX2OT*; chromosome 3: 182810845–182941699. *SOX2* region is shown by an arrow. The samples are classified based on the estrogen receptor status. D and E) Expression of *SOX2* and *SOX2OT* respectively in ER+ (n = 595) and ER− (n = 176) breast cancer samples (data derived from TCGA). Mann-Whitney rank sum test showed that these genes were expressed differently according to estrogen status: *p* values of <0.05 and <0.005 were calculated for D and E respectively.

Regulation of *SOX2* expression is poorly understood. An area located between positions −528 and +238 from the transcription start site is considered as the core proximal promoter region [26]. Additionally, an upstream enhancer centered between −3444 and −3833 of the transcription start site has an active role in controlling expression of *SOX2* in the reprogramming of oligodendrocyte precursors [27] (Figure 1A) and of pluripotent stem cells [28]. It has been shown the enhancer is activated upon sphere formation [25] in breast carcinoma cells, suggesting that reactivation of *SOX2* expression upon sphere formation may be controlled at the promoter level, in the same way as it is activated in pluripotent stem cells.

Genome-wide studies indicate that most of the human genome is transcribed, although only 1–2% of transcripts have protein-coding capacity [29], [30], [31]. Many of these transcripts are classified as long non-coding RNAs (lncRNAs) [29], [32]. Most studies have shown that lncRNAs genes are poorly conserved compared to protein-coding genes [33], [34]. Conservation analysis of lncRNAs based on 50-nt window size revealed that many lncRNAs retain patches of higher conservation, possibly representing interaction sites with RNA-binding proteins [34]. Additionally, some lncRNAs appear to be highly conserved among eukaryotes. The *SOX2* gene lies in an intron of a long multi-exon non-coding RNA gene that is transcribed in the same orientation (Figure 1B). This transcript in humans is named *SOX2OT* or “*SOX2 overlapping transcript*” and shares 88% identity with orthologous sequences in mouse [35]. *SOX2OT* has been postulated to participate in *SOX2* regulation [36]. It has been proposed that *SOX2OT* has a role in processes related to *SOX2* transcription, acting as an enhancer [36]. Recently, two novel splice variants of *SOX2OT* have been identified in esophageal squamous cell carcinoma and concordant expression of *SOX2* and *SOX2OT* was observed [37]. Currently, very little is known regarding *SOX2OT* expression in breast cancer and its role in tumor initiation or progression. In this report we show that *SOX2OT* positively regulates *SOX2* expression, and the ectopic expression of *SOX2OT* in the breast cancer cell line MDA-MB-231 reduces the proliferation rate and increases anchorage independent growth.

## Materials and Methods

### Chemicals and reagents

MammoCult medium was purchased from Stem Cell Technologies. Anti-SOX2 antibody was purchased from Cell Signaling Technology (catalogue number 2748). Goat anti-Rabbit IgG2a antibody was from Santa Cruz Biotechnology (catalogue number SC-2054).

*SOX2OT* and control vectors (EX-hLUC-M90) were purchased from GeneCopoeia. The *SOX2OT* construct was custom made using *SOX2OT* splice variant with accession number NR_004053.

### Cell culture

Breast cancer cell lines as well as normal breast epithelial cell line MCF10A were cultured according to ATCC recommendations or as described previously [38]. Derivatives of MCF-7 cells were cultured as previously described [39]. MammoCult medium were used for suspension culture.

### Cell suspension culture

Cells of the MCF-7 parental line and derivative sub-lines as well as MDA-MB-231 were trypsinized from monolayer culture, and suspensions containing 2×10^6^ cell seeded in non-tissue culture treated T-25 flasks (Nunc catalogue number 169900). Cells were grown in MammoCult medium, at 37°C in 5% CO_2_ in air. All experiments were performed at least two times.

### Mouse mammary fat pad model

The orthotopic human breast cancer xenograft model was generated as previously described [40]. Basically, 2×10^6^ MDA-MB-231-luc-D3H2LN cells (from Caliper Life Sciences) in 50 µl of 50% Matrigel (BD Biosciences), 50% serum free alpha-MEM medium were inoculated into the 4^th^ right mammary fat pad of female NIH III nude mice (bred at the University of Auckland). The primary tumors were collected 8 weeks after inoculation for quantitative measurement of gene expression by quantitative real time PCR (qRT-PCR). Animal studies were performed in accordance with the approval (CR830) from the Animal Ethics Committee of the University of Auckland.

### RNA extraction, reverse transcription, quantitative PCR and PCR

Total RNA from cultured cells or tumor tissues was purified using Trizol (Invitrogen) and treated with DNase I (Sigma) according to the manufacturer's instructions. Oligo-dT or random hexamer were used to reverse transcribe 1 µg of RNA with SuperScript III Reverse Transcriptase (Sigma) according to the manufacturer's instructions. cDNA preparation and qRT-PCR analysis were performed as described previously [41]. For non-quantitative expression analysis, cDNA was amplified by PCR for 35 cycles at 60°C as annealing temperature and amplified products were visualized after electrophoresis in a 1–2% agarose gel.

Quantitative PCR was performed with a final 1 in 20 dilution of cDNA, 8 µM primers and Sybr Green MasterMix (Invitrogen). For normalization of transcript expression levels, human glyceraldehyde 3-phosphate dehydrogenase (*GAPDH*) and hypoxanthine-guanine phosphoribosyltransferase (*HPRT*) transcripts were used as internal controls. The average expression of internal control was used to calculate the relative gene expression.

Table S1 contains the list of primers used.

### Western Blotting

Total protein was collected from cultured breast cancer cells. Cells were lysed in buffer containing 60 mM Tris-HCl, pH 6.8, 2% SDS, and 20% glycerol, and protein quantitated by BCA assay. Cell lysates containing 25 µg of protein were separated by SDS-PAGE, and transferred to PVDF membranes (Millipore). Membranes were immunoblotted with antibodies against SOX2 (1∶500 for anti-SOX2) and β-actin (1∶2500) (Sigma), and antibody visualized using SuperSignal West Pico (Thermo Scientific, Waltham, MA for β -actin) or ECL plus (for SOX2) (Thermo Scientific). Bound antibody was visualized using the chemiluminescence detection system (Fujifilm Las-3000).

### Ectopic Expression Of *Sox2ot*

Constructs overexpressing *SOX2OT* and control empty plasmid (vector) (Ex-NEG-M90) were purchased from GeneCopoeia. Both plasmids express GFP to enable detection of transfected cells. Breast cancer cells (MDA-MB-231) were transfected with 5 µg of DNA and Lipofectamine Plus (Invitrogen) according to the manufacturer's instructions. Two biological replicates for each construct were made, the transfected cells treated with puromycin and selected on the basis of GFP expression by fluorescence-activated cell sorting (FACS) as previously described [42]. The sorted cells were maintained in the presence of puromycin.

### Cell Proliferation Assays

The sulforhodamine B colorimetric (SRB) assay, which is based on the measurement of cellular protein content, was used to measure cell density [43]. Briefly, 3000 cells were plated in 96 well tissue culture plates, and were harvested after 1, 3, 5 and 7 days. Cell proliferation was also measured using a thymidine incorporation assay in which 3000 cells per well were seeded in 96 well plates that were tissue culture-treated for monolayer culture and incubated for 3 days. Alternatively, 10,000 cells per well were seeded in 96 well plates (Corning Costar Ultra-Low attachment) for 6 days suspension culture. Briefly, 0.04 µCi of ^3^H-thymidine was added to each well and the cultures incubated for 5 h, after which the cells were harvested on glass fiber filters using an automated TomTec harvester. Filters were incubated with Betaplate Scint and thymidine incorporation was measured using a Trilux/Betaplate counter.

All experiments were done using at least triplicate wells, and performed on at least two separate occasions.

### Cell Cycle Analysis

Cells were grown as monolayers in flasks and in the presence or absence of the mitotic poison paclitaxel (200 nM) for 24 h prior to cell cycle analysis. Non-adherent cells were discarded by aspiration and the attached cells were trypsinized and counted. 10^6^ cells were washed with cold PBS, then with cold PBS containing 1% FCS (twice), and fixed with ice-cold methanol overnight at −20°C. The cells were centrifuged at 110 g for 5 min, washed in 1 mL cold PBS containing 3% FCS, and resuspended in the same buffer. The cells were treated with RNase (100 µg/mL) and propidium iodide (PI) (100 µg/mL) for 30 min at room temperature. DNA content was determined by PI fluorescence using a BD FACSVantage cytometer and a total of 30,000 acquired events. The results were analyzed using ModFit LT (Verity Software House).

### Assay For Anchorage-Independent Proliferation

The 96 well plates were pre-coated with 40 µl of preheated 0.15% agarose solution. Pre-mixed 2× medium and 0.4% agarose to give the final concentration of 0.2% were prepared. A total of 9000 cells were added in 1× medium. When the top layer of cells containing agarose had gelled, 50 µl of cell culture medium was added to each well. Cells were cultured for 2 weeks with a change of medium every three to four days. Cells were visualized using 0.5% crystal violet, and colonies of 5 cells or more were counted under phase contrast microscope (5× magnification). All experiments were done using at least 10 replicates, and performed at least three times.

### Statistical Analysis

Statistical analysis was performed using SigmaPlot. Data were analyzed using either one-way ANOVA coupled with multiple comparisons versus treatment control applying the Holm-Sidak method correction, where *p*<0.05 denotes a statistically significant difference. Correlation analysis was performed with Spearman's rank correlation coefficient (*R*) and statistical significance (*P*). Values of *p*<0.05 were considered to be statistically significant.

## Results

### Expression Of *Sox2* And *Sox2ot* In Breast Cancer Samples From The Cancer Genome Atlas (tcga) Data Set

We analyzed the genome-wide RNA transcript profile from TCGA (breast invasive carcinoma gene expression) by RNAseq data set (TCGA_BRCA_exp_HiSeqV2-2013-12-18) including 1106 samples from breast cancer patients, and found distinct differences in the expression patterns of *SOX2* and *SOX2OT* in breast cancer samples (Heat map, Figure 1C). This data confirms expression of *SOX2* and *SOX2OT* are positively correlated (Spearman Rank Order Correlation coefficient, *r* = 0.219; *p* = 2.23×10^−13^) showing higher expression of both *SOX2* and *SOX2OT* in estrogen receptor positive (ER+) compared to ER− tumors. Statistically significant differential expression of *SOX2* (*p* = 0.001) and *SOX2OT* (*p*<0.001) was observed in 595 ER+ and 176 ER− samples from the same data set using Mann-Whitney Rank Sum test (Figure 1D, 1E).

### Expression Analysis Of *Sox2* And *Sox2ot* In Breast Cancer Cell Lines

**Figure 2:**
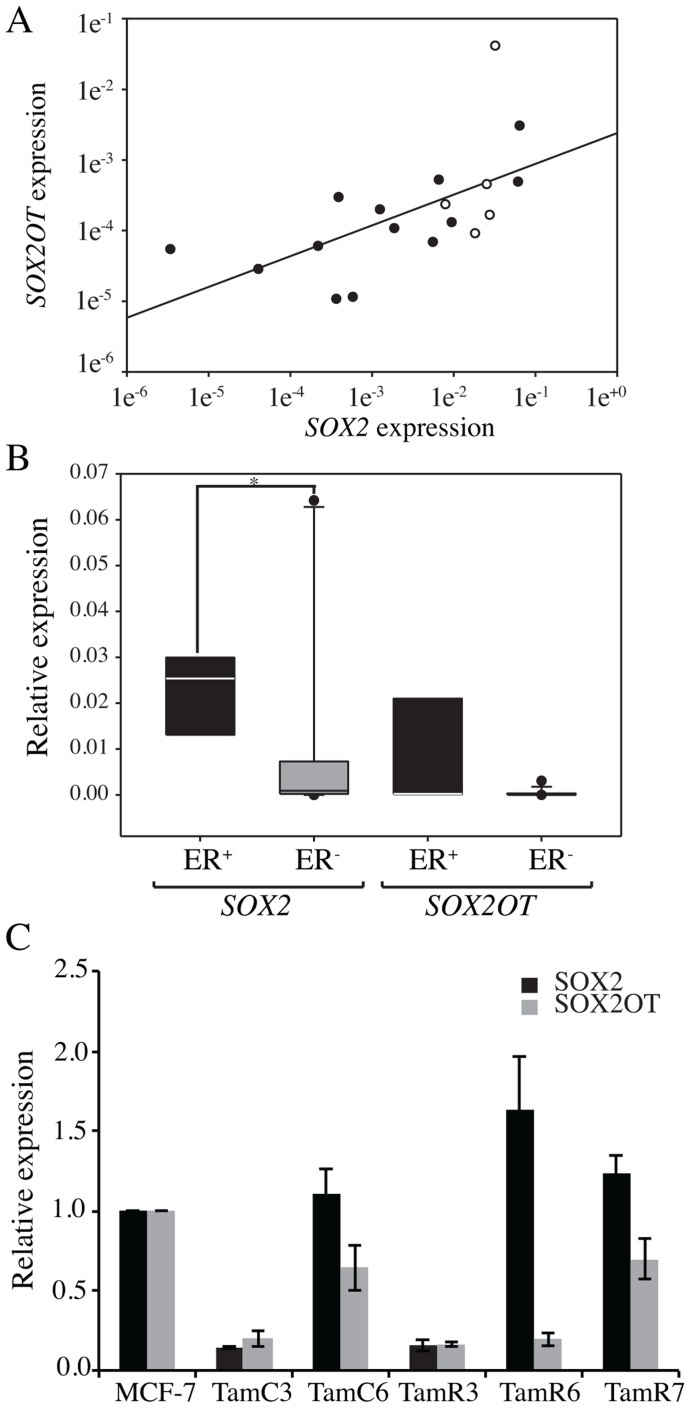
Expression of SOX2 and *SOX2OT* in different breast cancer cell lines. A) The expression of *SOX2* and *SOX2OT* relative to *HPRT* and *GAPDH* in 18 breast cell lines measured by qRT-PCR. Scatter plot showing the expression of *SOX2* and *SOX2OT*. Black and white circles represent ER− and ER+ cell lines. B) Box plot indicating expression of *SOX2* and *SOX2OT* in ER+ and ER− breast cancer cell lines. * represents *p* value <0.05. C) Expression of *SOX2* and *SOX2OT* in five MCF-7 sub-lines relative to the MCF-7 parental line was measured by qRT-PCR. Error bars represent standard deviations of three technical replicates.

We next examined the relative expression of *SOX2* and *SOX2OT* in 18 breast cell lines by qRT-PCR (Figure 2A, and Figure S1). *SOX2* was expressed in all of the ER+ cell lines, as compared to six out of twelve ER− cell lines; widely different levels of expression were detected. *SOXOT* showed lower levels of expression than its protein-coding counterpart, as is the case for many lncRNAs [44], and a strong positive correlation between the expression of the two genes was observed by Spearman rank order correlation (*r* = 0.748; *p* = 12×10^−5^) (Figure 2A). As with TCGA data, significantly higher *SOX2* expression was observed in ER+ cell lines (*p* = 0.03). However, no significant difference in *SOX2OT* expression was observed between ER+ and ER− cell lines (Figure 2B).

### Tamoxifen-Resistant Breast Cancer Cells Express Similar Levels Of *Sox2*

It has been reported that *SOX2* expression is associated with the promotion of tamoxifen resistance in breast cancer, and that ectopic expression of *SOX2* contributes to tamoxifen resistance in the MCF-7 breast cancer cell line [45]. We therefore examined the expression of *SOX2* and *SOX2OT* in a series of tamoxifen-resistant MCF-7 breast cancer sub-lines that had been cultured for prolonged periods either in the absence of estrogen or in the presence of the antiestrogen tamoxifen [39]. Expression analysis confirmed the slightly higher levels of expression of *SOX2* in three tamoxifen resistant sub-lines TamC6, TamR6 and TamR7 (Figure 2C). Unexpectedly, two other cell lines, TamC3 and TamR3, showed reduced *SOX2* expression. Concordant expression of *SOX2* and *SOX2OT* was not observed in these sub-lines.

### Suspension Culture

**Figure 3:**
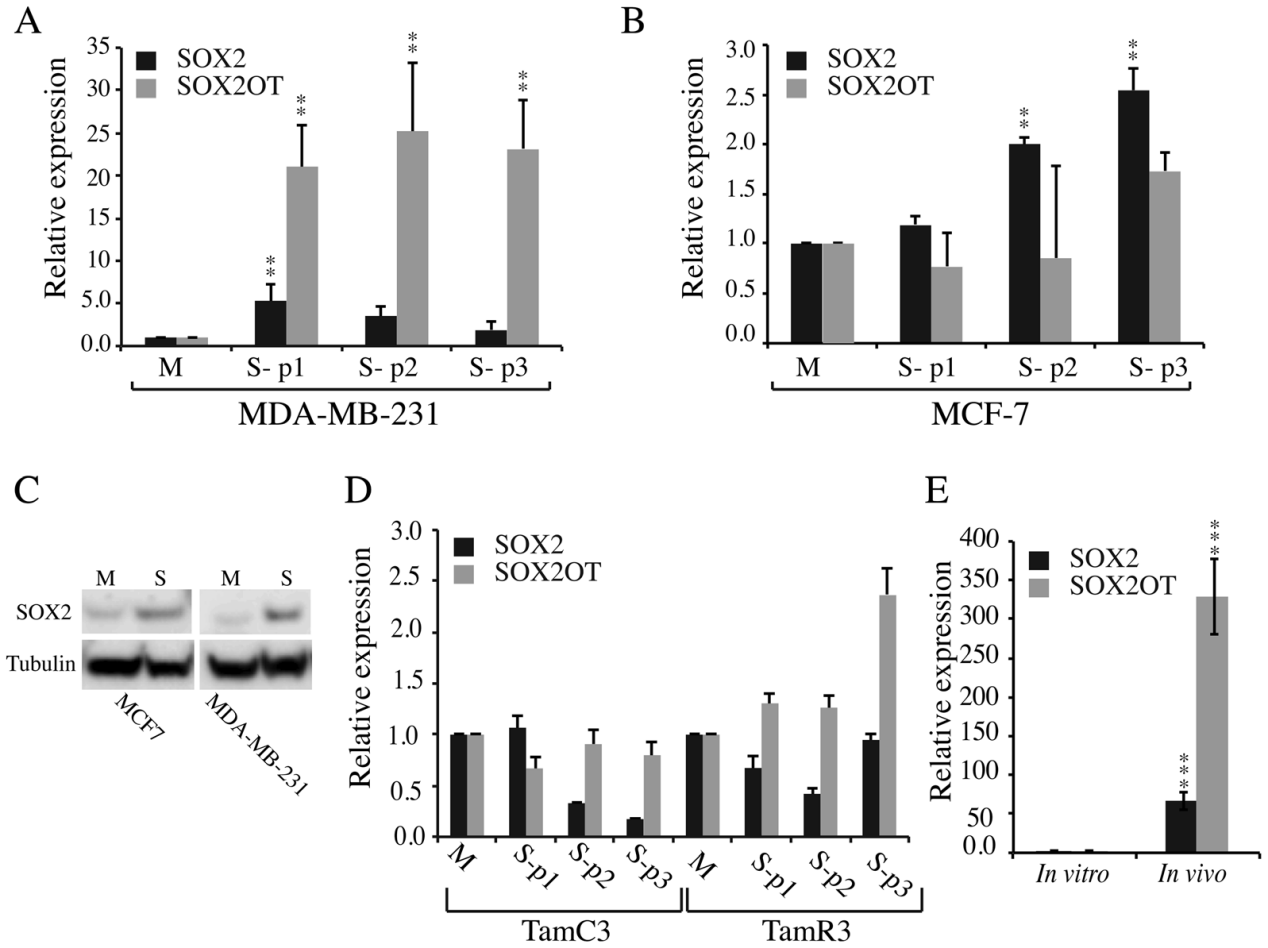
Expression on *SOX2* and *SOX2OT* in breast cancer cell lines grown in suspension culture. A and B) Expression of *SOX2* and *SOX2OT* in MDA-MB-231 (A) and MCF-7 (B) breast cancer cell lines cultured in suspension relative to the cells grown as monolayer was measured by qRT-PCR. Three consecutive passages of cells were grown in suspension as S-p1 to S-p3 respectively. M represents the expression in monolayer culture. Error bars represent the standard error of the mean of three biological replicates. C) Western blot analysis showing expression of SOX2 in MCF-7 and MDA-MB-231 cells grown as monolayers and in suspension. D) Expression of *SOX2* and *SOX2OT* in TamC3 and TamR3 derivatives of MCF-7 grown in suspension relative to MCF-7 monolayer culture measured by qRT-PCR. Error bars represent standard deviations of three technical replicates. E) Expression of *SOX2* and *SOX2OT* in monolayer MDA-MB-231-luc-D3H2LN cells (*in vitro*) and the same cells grown as orthotopic xenografts (*in vivo*) were measure by qRT-PCR. The error bars are standard error of the mean of six individual tumors. *, **, and *** represent *p* values of <0.05, 0.01 and 0.001 respectively.

Growth in suspension culture is commonly used to examine both normal and neoplastic cells for clonogenic growth potential. Sphere formation has been reported for many established breast cancer cell lines and there is some evidence to suggest that spheres may be enriched for cells with cancer stem cell phenotypes [38],[46],[47],[48]. To examine the influence of culture conditions on the relative expression of *SOX2* and *SOX2OT*, MCF7 and MDA-MB-231 cell lines were grown either as monolayers (M), or in suspension cultures (S). MCF-7 cells showed higher *SOX2* expression levels than did MDA-MB-231 cells when cultured as monolayers (Figure S1). We observed some significant increases in expression of *SOX2* or *SOXOT* in both MCF-7 and MDA-MB-231 cell lines when cultured as spheres compared to adherent monolayers (Figure 3A and B). *SOX2OT* expression was up-regulated relative to *SOX2* in the triple negative MDA-MB-231 cell line but down-regulated in the estrogen receptor positive MCF-7 cell line (Figure 3A and B). To determine whether up-regulation of *SOX2* mRNA expression led to higher protein levels in these cells, immunoblot analysis was performed using the first passage of cells propagated in suspension culture, confirming the up-regulation of SOX2 protein in this culture in both MCF-7 and MDA-MB-231 cells (Figure 3C).

We also compared *SOX2* and *SOX2OT* expression in our MCF-7 tamoxifen-resistant sub-lines TamC3 and TamR3 [39] cultured as monolayers or in suspension. In contrast to the MCF-7 parental line, *SOX2* expression was down-regulated in suspension culture while *SOX2OT* was up-regulated in the third passage of suspension cultured TamR3 (Figure 3D).

### Expression Of *Sox2* And *Sox2ot* In Xenografts

To examine whether *SOX2* and *SOX2OT* are up-regulated in xenografts, we evaluated their expression using a MDA-MB-231 variant (MDA-MB-231-luc-D3H2LN) [40]. We compared the level of expression of *SOX2* and *SOX2OT* in MDA-MB-231-luc-D3H2LN cells grown in monolayer with those injected to the mouse fat pad and grown for 8 weeks. The expression of *SOX2* and *SOX2OT* (Figure 3E) was found to be significantly up-regulated in tumor xenografts.

### Ectopic Expression Of *Sox2ot* Up-Regulates *Sox2* Expression

The MDA-MB-231 cell line was selected for study because its basal expression of *SOX2OT* was very low. We attempted to identify different isoforms of *SOX2OT* in MDA-MB-231 and MCF7 cells grown in suspension culture and identified one isoform in both cell lines, as shown in Figure S2.

**Figure 4:**
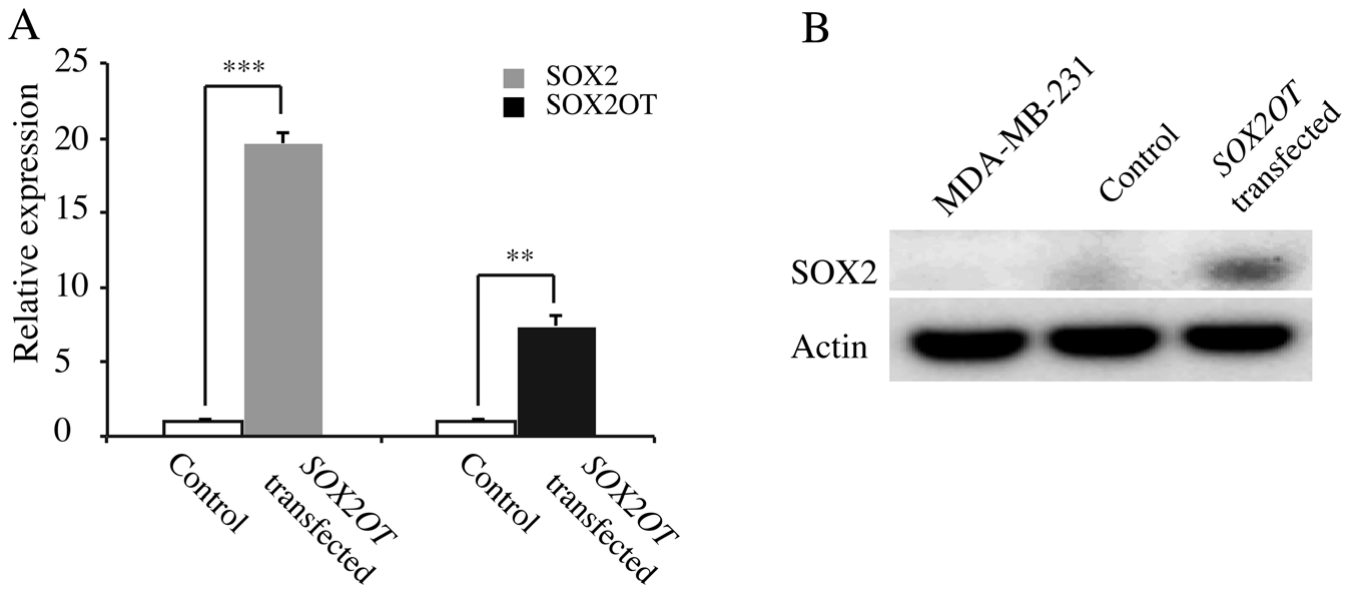
Ectopic expression of *SOX2OT* in MDA-MB-231 cells. A) Relative expression of *SOX2* and *SOX2OT* measured by qRT-PCR in MDA-MB-231 cells transfected with control vector and plasmid containing a *SOX2OT* gene (NR_004053.3). The bar graphs show the fold change relative to control vector. ** and *** represent *p* values of <0.01 and 0.001 respectively. B) Western blot analysis showing the over-expression of *SOX2* in cells transfected with vector containing the *SOX2OT* gene.

The expression of *SOX2* and *SOX2OT* was measured by qRT-PCR in MDA-MB-231 cells transfected with control vector or *SOX2OT* overexpression vector (Figure 4A). The relative expression of *SOX2OT* was increased by about 8-fold in *SOX2OT* transfected cells in comparison to control vector, while 20-fold up-regulation was observed in *SOX2* expression. This result provides evidence for a positive regulatory role of *SOX2OT* in *SOX2* transcription.

SOX2 protein was also induced in parallel with the up-regulation of its mRNA in *SOX2OT*-transfected MDA-MB-231 cells (Figure 4B). These data confirm that the up-regulation of *SOX2OT* transcript leads to higher abundance of SOX2 protein in ectopic *SOX2OT* expressing cells. However, the expression of two other stem cell marker genes, *OCT4* and *NANOG*, was not significantly altered in the *SOX2OT*-transfected MDA-MB-231 cells (Figure S3). Expression of *NANOG* in these cells was very low while *OCT4* had a higher level of expression.

### *Sox2ot* Expression Reduces Proliferation

**Figure 5:**
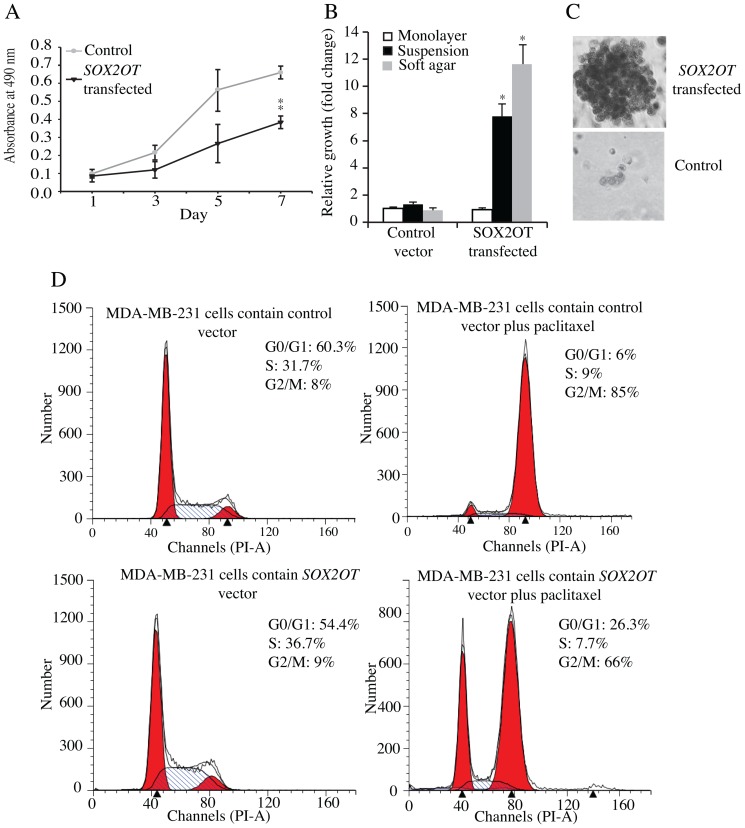
Effect of ectopic expression of *SOX2OT* on cell growth. A) The proliferation rates of cells transfected with control vector and plasmid containing the *SOX2OT* gene were measured after 1, 3, 5 and 7 days of culture. B) Proliferation rates of cells transfected with control vector or plasmid containing the *SOX2OT* gene, in monolayer and suspension culture, as well as in soft agar. * and ** represent *p* values of <0.05 and 0.01 respectively. C) Image of MDA-MB-231 cells grown in soft agar for two weeks. The top panel represents the colony form in cells with ectopic expression of *SOX2OT*. The bottom panel represents cells containing the control vector. D) Effect of ectopic expression of *SOX2OT* on cell cycle distribution. The cells were treated with and without paclitaxel for 24 h and stained with PI. Following FACS analysis, DNA histograms were analyzed using ModFit LT. The data are representative examples for duplicate tests.

MDA-MB-231 cells ectopically expressing *SOX2OT* showed significantly slower growth rate than cells transfected with the control vector, as measured by SRB assay on monolayer culture (Figure 5A). Proliferation of *SOX2OT*- and control vector-transfected MDA-MB-231 cells was also studied using thymidine incorporation assay in either monolayers or suspension cultures in Ultra-Low attachment 96-well plates. The *SOX2OT* transfected cells showed a significantly higher proliferation rate in suspension culture, suggesting that *SOX2OT* overexpression can lead to increased anchorage independent cell growth. (Figure 5B black bars).

To further investigate anchorage independent growth, *SOX2OT* or control vector transfected cells were examined using a soft agar colony formation assay. The number of colonies formed in *SOX2OT* transfected MDA-MB-231 cells significantly increased, as compared with control cells (Figure 5B). *SOX2OT* overexpression not only increased the number of colonies, but also induced a significant increase in colony size with cells expressing higher levels of *SOX2OT* (Figure 5B and C).

### *Sox2ot* Effects On The Cell Cycle

To investigate whether ectopic expression of *SOX2OT* altered cell cycle progression, control and *SOX2OT*–overexpressing cells were grown as monolayer adherent cultures for 24 h in the presence of paclitaxel to induce mitotic arrest and to prevent cell division. The cell cycle distribution was then determined by flow cytometry. The progression of cells over 24 h from G1- and S-phase to G2/M-phase cells was used as a measure of cell cycling. The progression to G2/M phase for control cells was 77% (8% to 85%) while that for *SOX2OT* overexpressing cells was 57% (9% to 66%), confirming that cell cycle progression was delayed by *SOX2OT* overexpression and consistent with the slower growth rate (Figure S4).

## Discussion

We show here that the ectopic expression of a long non-coding RNA, *SOX2OT*, dramatically up-regulates expression of the developmental gene *SOX2*. The *SOX2* gene encodes an HMG domain-containing transcription factor that is expressed in various phases of embryonic development in a manner linked to cell-type specification and cellular differentiation [1],[49]. Like many other developmental regulatory factors, the dysregulated expression of *SOX2* genes has been implicated in a number of human cancers [16],[17],[22],[50],[51]. Poorly differentiated tumors show higher levels of expression of embryonic stem cell markers including *SOX2* [21]. *SOX2* is also overexpressed in human breast cancer tissue and cell lines, and its levels are correlated with tumor grade [10]. Abnormal expression of *SOX2* affects cell fate, proliferation, cancer progression and the control of key transcriptional processes in stem cells [52] but little is known about its regulation. Our results therefore provide an important clue as to how *SOX2* expression might be controlled in cancer tissue. Previously Chen *et al.* [10], analyzed gene expression in a panel of breast cancer cell lines and primary tumors, and both gain- and loss-of-function experiments indicated that *SOX2* contributes to breast cancer cell proliferation and tumorigenic properties *in vitro* and *in vivo*. They also showed that at the cellular level, *SOX2* promotes cell cycle progression. In contrast, Cox *et al.* [11], reported significant dose-dependent reductions in cell number upon overexpression of *SOX2* in MCF-7 and MDA-MB-231 breast cancer cell lines. These results, in conjunction with our own showing reduced cell proliferation following overexpression of *SOX2* (Figure 5A), suggest a model according to which *SOX2* expression has a biphasic effect on cell proliferation, stimulating it at lower levels and inhibiting it at high levels. This result may also suggest that *SOX2* expression affects proliferation differentially according to whether proliferation is on monolayer culture or in suspension. This result also suggests that *SOX2OT* can act in *trans* to induce *SOX2* expression.

The induction of *SOX2* by ectopic expression of *SOX2OT* establishes a possible signaling pathway but does not prove that this pathway is important in human breast cancer. To address this question, we compared *SOX2* and *SOX2OT* expression in a large collection of breast cancer samples from TCGA. A weak (*r* = 0.219) but highly significant (*p* = 2.23×10^−13^) correlation between expression of *SOX2* and *SOX2OT* was found (Figure 1), emphasizing the potential importance of this signaling pathway in human breast cancer. A further positive correlation (*r* = 0.761) was found (Figure 2A) between the expression of the two genes in cell lines (*p* = 2×10^−7^). This cell line data also showed higher expression of *SOX2* and *SOX2OT* in ER+ than in ER− samples (Figure 2B). However, our data differ from a previous report that found an inverse association of *SOX2* expression with ER and PR [22]. This may suggest more complex associations of *SOX2* and ER in different breast cancer subtypes, since Rodriguez-Pinilla *et al.* (2007) examined tumors with *BRCA1* mutations and sporadic basal-like breast carcinomas while the samples from TCGA data are more variable with respect to sub-type. We could not detect any association between *SOX2* and *SOX2OT* expression in basal and luminal cell lines. Our data with breast cancer cell lines are in agreement with TCGA data, and add to broader evidence obtained during recent years that lncRNAs are major regulatory molecules in normal cellular development and disease progression.

It has been proposed that *SOX2* expression is induced when cells are propagated in suspension culture [23]. We therefore investigated the relative expression of *SOX2* and *SOX2OT* in suspension cultures. Interestingly, *SOX2OT* was up-regulated in both MCF7 and MDA-MB-231 cells grown in suspension, and the extent of *SOX2OT* up-regulation was more pronounced in the triple negative MDA-MB-231 cell line. The cells grown as orthotopic breast xenograft tumors also showed significant up-regulation of both *SOX2* and *SOX2OT* in the primary xenograft site. These data confirm and extend previous results for suspension cultures.

Enhanced *SOX2* expression was reported to confer tamoxifen resistance in the MCF-7 cell line and *SOX2* expression is also higher in breast cancer tissue taken from patients after endocrine therapy failure [45], suggesting that the development of tamoxifen resistance is associated with activation of the pathway leading to *SOX2*-expression [45]. We have previously developed a series of tamoxifen-resistant sub-lines of the MCF-7 cell line using conditions that mimic the development of clinical tamoxifen resistance [39],[42],[53],[54],[55],[56],[57], and it was of interest to determine whether these also exhibited increased *SOX2* expression. As shown in Figure 2C, three lines (TamC6, TamR6 and TamR7) did indeed show a tendency to increase *SOX2* expression. However, two (TamC3 and TamR3) showed lower expression, indicating that *SOX2* expression is not always coupled to tamoxifen resistance. All lines except TamC6 showed reduced progesterone receptor and increased estrogen receptor expression [54] suggesting that steroid receptor expression was not closely coupled to *SOX2* expression. However, TamC3 and TamR3 exhibit strongly reduced mTOR signaling [39], together with decreased glycolysis rates and increased sensitivity to cytotoxic drugs (unpublished data), suggesting that *SOX2* expression is coupled to mTOR pathway utilization. This is consistent with the results of Corominas-Faja *et al* suggesting that low utilization of the mTOR pathway is associated not only with low *SOX2* expression but also with reduced stem cell properties [58], and inhibition of mTOR disrupts pluripotency and represses *SOX2* expression [59]. It is possible that decreased mTOR pathway utilization in TamC3 and TamR3 has contributed to the repressed *SOX2* and *SOX2OT* expression observed. Hence, we compared the ability of these lines to form mammospheres. The resistant line TamR3 was found to be less efficient than MCF-7 parental cells in mammosphere formation and they formed only compact spheroids (data not shown). This supports the hypothesis that *SOX2* expression, mTOR pathway utilization and stem cell functions have mechanistic relationships.

In conclusion, an earlier observation that the *SOX2OT* locus has complex architecture and is important during vertebrate development in a number of systems [36] has been extended in this study to a series of human breast cancer lines. The previous suggestion that *SOX2OT* plays a role in regulating chromatin structure [36] has been extended, to demonstrate a specific effect of *SOX2OT* on *SOX2* regulation, using both expression arrays from human breast cancers, a series of breast cancer cell lines and a single line exhibiting ectopic expression of *SOX2OT*. The results add to the growing picture that emphasizes the importance of non-coding RNA in tumor cell properties.

## Supporting Information

Figure S1 **Relative expression of** ***SOX2*** **and** ***SOX2OT*** **in different breast cancer cell lines.** A and B) Relative expression of *SOX2* (A) and *SOX2OT* (B) to *GAPDH* and *HPRT* were measured by qRT-PCR in different breast cancer cell lines. The error bars are standard error of mean of three biological replicates. (TIF)

Figure S2 **Different isoforms of** ***SOX2OT*** **.** A) Schematic diagram showing two different isoforms of *SOX2OT*. The boxes and horizontal lines represent the exons and introns respectively. The arrows on top demonstrate the location of primers used in PCR. B) Gel electrophoresis of PCR products using different primers and cDNA derived from MDA-MB-231 cells grown in suspension culture. Lanes 1 to 4 are the combination of F2+R1, F3+R1, F4+R1 and F5+R1 respectively. Expected sizes for lanes 1, 2 and 4 are 205, 754 and 312 bp respectively. C) Nested PCR using F2+R2 as primers and the product from lane 2 in panel B as template. Expected size for this PCR product is 100 bp. Using F4 as forward primer in combination of R1 or R2 in PCR reaction did not amplify any product. (TIF)

Figure S3 **Expression analysis of stem cell-like markers in cells with ectopic expression of** ***SOX2OT*** **.** The expression of *SOX2*, *SOX2OT*, *OCT4* and *NANOG* in MDA-MB-231 cells transfected with control vector and vector containing *SOX2OT* were measured by qRT-PCR. The bar graphs show the fold change relative to control vector. The error bars represent standard errors from two biological replicates and three technical replicates. *p*-values for *SOX2*, *SOX2OT*, *OCT4* and *NANOG* were <0.001***, 0.007**, 0.13 and 0.85 respectively. (TIF)

Figure S4 **Cell cycle analysis in cells with ectopic expression of** ***SOX2OT*** **.** MDA-MB-231 cells were treated under different experimental conditions and were collected for cell cycle profile analysis by flow cytometry. A) The percentage of cells in G0/G1, S, and G2/M phases is shown. B) Ratio of G1/S populations calculated in cells transfected with empty vector and plasmid containing the *SOX2OT* gene in MDA-MB-231 cells treated with paclitaxel. Error bars represent standard deviations of two biological replicates. (TIF)

Table S1 List of primers used in these experiments. The sequences are 5′ to 3′. (DOCX)
