# Supplementary figures and images for: Emerging Role of Long Non-Coding RNA SOX2OT in SOX2 Regulation in Breast Cancer
Source: PLoS One. 2014 Jul 9;9(7):e102140. doi: 10.1371/journal.pone.0102140 (PMC4090206; doi:10.1371/journal.pone.0102140)

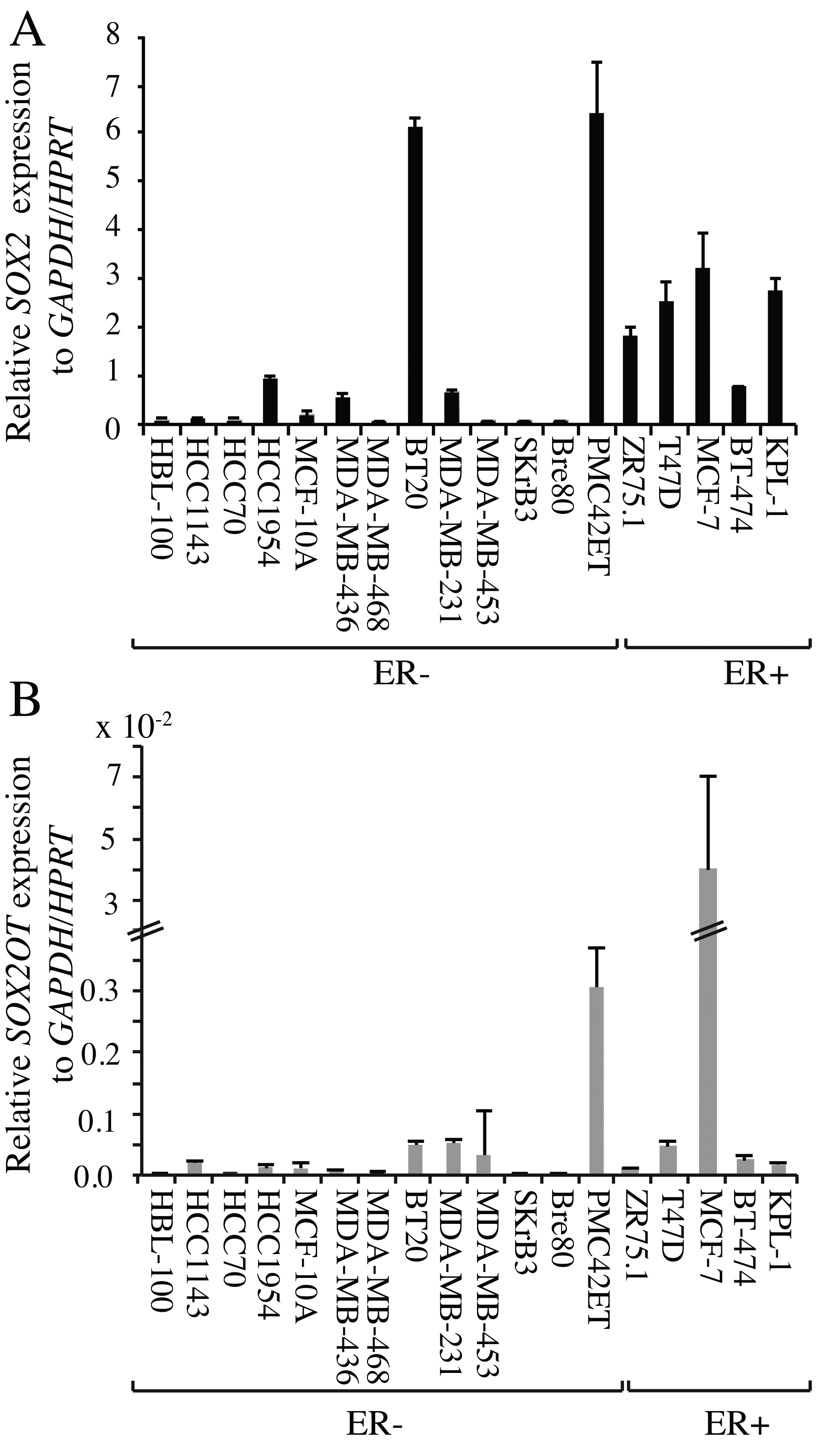

Supplement: Figure S1 — (TIF) [file pone.0102140.s001.tif]

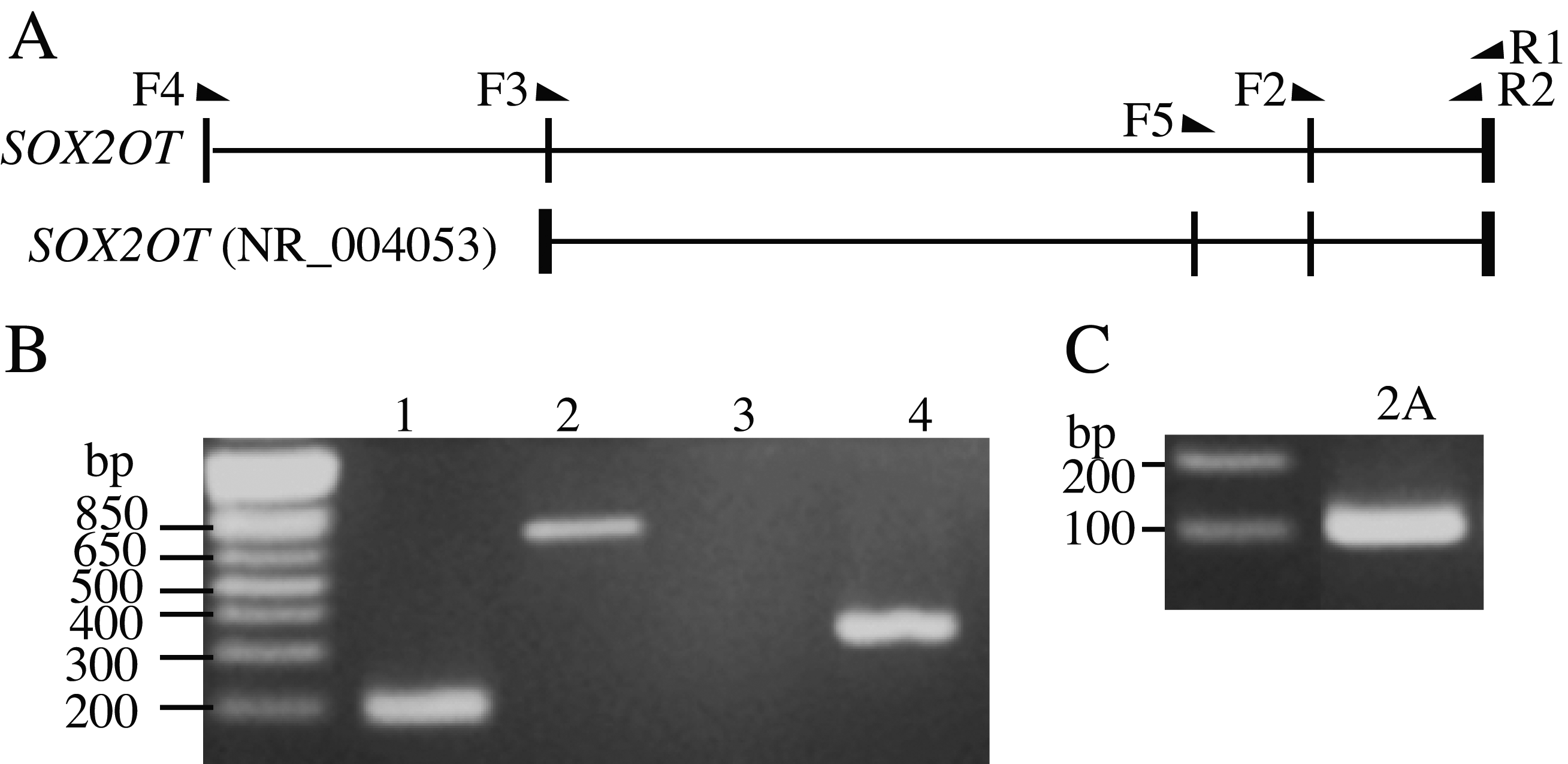

Supplement: Figure S2 — (TIF) [file pone.0102140.s002.tif]

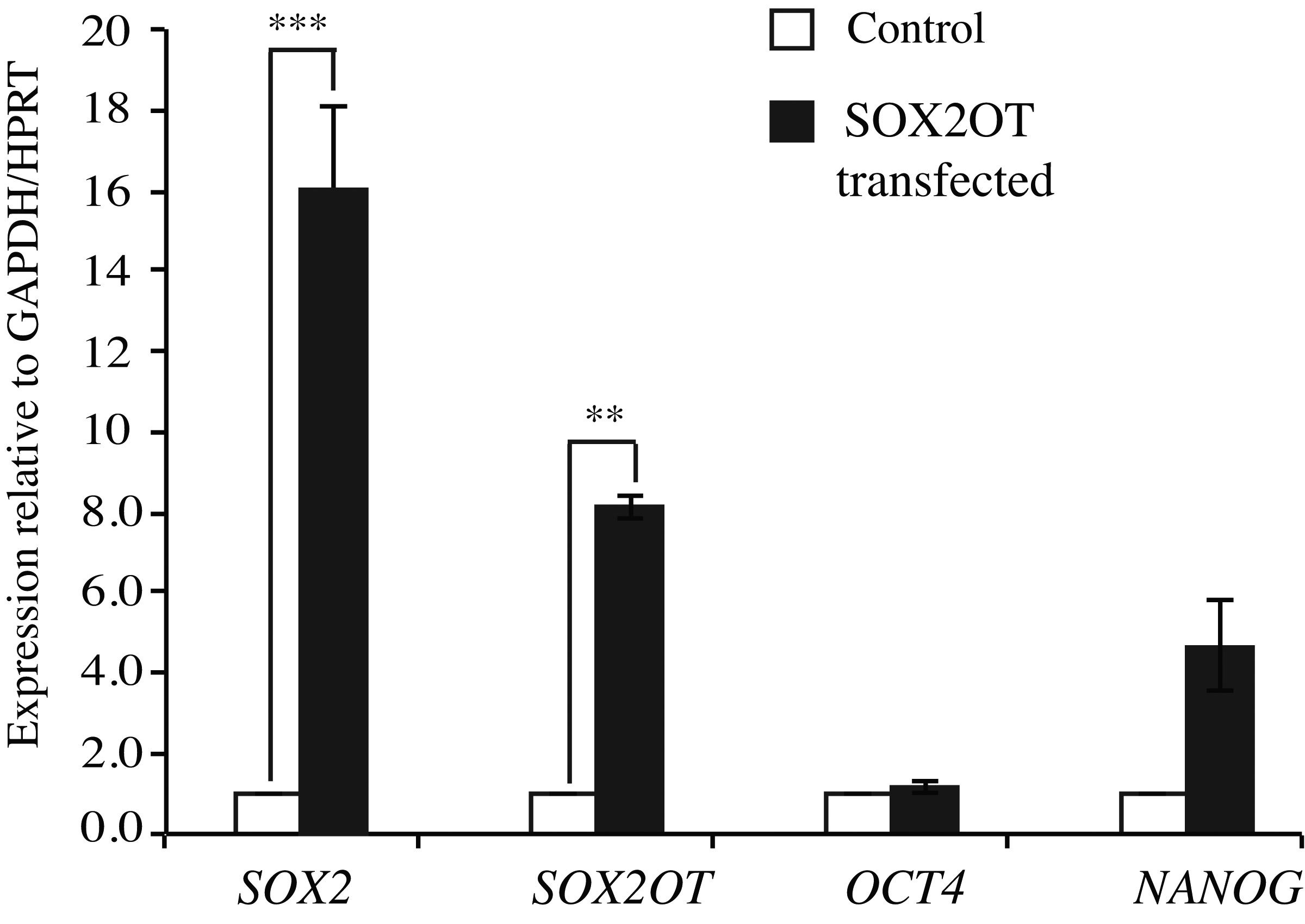

Supplement: Figure S3 — (TIF) [file pone.0102140.s003.tif]

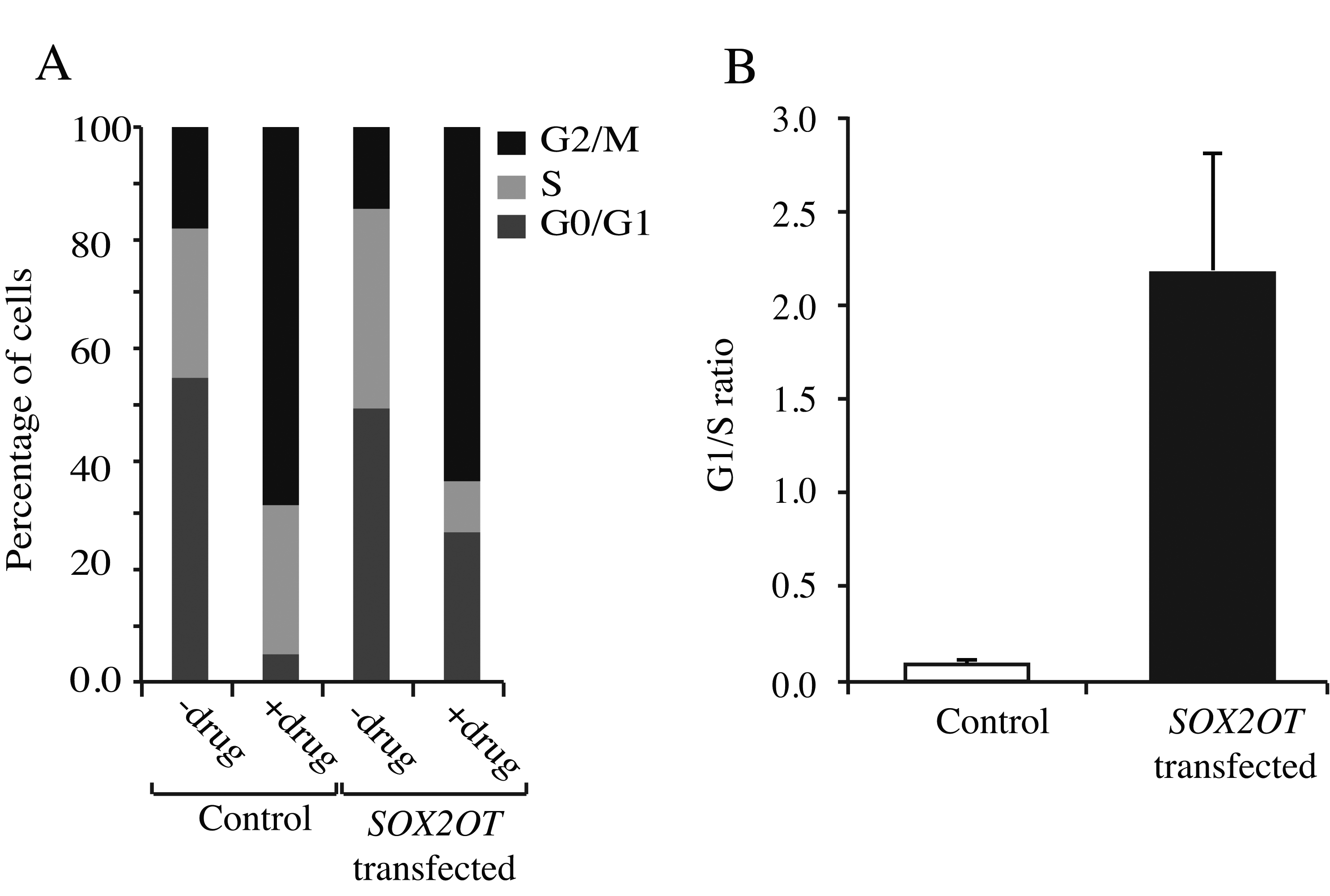

Supplement: Figure S4 — (TIF) [file pone.0102140.s004.tif]
