# Supplementary material for: Emerging Role of Long Non-Coding RNA SOX2OT in SOX2 Regulation in Breast Cancer
Source: PLoS One. 2014 Jul 9;9(7):e102140. doi: 10.1371/journal.pone.0102140 (PMC4090206; doi:10.1371/journal.pone.0102140)
Supplement: Table S1 — (DOCX) [file pone.0102140.s005.docx]

| SOX2-F | AACCCCAGATGCACAA CTC |
| --- | --- |
| SOX2-R | GCTTAGCCTCGTCGATGAAC |
| SOX2OT-F2 | CAAAAGAGAAGCCAGGCAAC |
| SOX2OT-R2 | AAGCCACGAGCCTTATCTTG |
| Sox2OT-F5 | TTTACCTGCCAATCAAACTGC |
| Sox2OT-F3 | CAGTCAGCTGGTCCCTCTTC |
| Sox2OT-F4 | AACTTCATTGATCGCCAAGG |
| SOX2OT-R1 | CTGGCAAAGCATGAGGAACT |
| HPRT-F | TGAGGATTTGGAAAGGGTGT |
| HPRT-R | GCACACAGAGGGCTACAATG |
| GAPDH-F | ACGGG AAGCTTGTCATCAAT |
| GAPDH-R | TGGACTCCACGACGTACTCA |
| OCT4-F | GTGGAGGAAGCTGACAACAA |
| OCT4-R | CTCCAGGTTGCCTCTCACTC |
| NANOG-F | CCTATGCCTGTGATTTGTGG |
| NANOG-R | CTTGACCGGGACCTTGTCTT |
